# Commemoration of 1000^th^ liver transplantation in Shiraz Center

**Published:** 2011-08-01

**Authors:** Ali Bahador

**Affiliations:** *Congress Executive Secretary*

The 10^th^ Congress of Iranian Society for Organ Transplantation (IRSOT 2011) was held in Shiraz in May 17–19, 2011. Most of the scientific staff of Iranian Transplantation wards and many colleagues who have been involving in transplantation from the Middle East, United States and other countries participated in this Congress.

During the Congress, on evening of May 18, in commemoration of 1000 liver transplantation in Shiraz, a group of people gather together to celebrate this great event. All Congress participants, chairmen of transplant wards, social and official managers of Shiraz and families of donors and recipients took part in this feast.

Dr. S. A. Malek-Hosseini, Chairman of Shiraz Organ Transplant Center briefly gave a report on history and progression of the Shiraz Transplant Center. The first liver transplant was performed in 1993, before deceased donor approval law in Iranian parliament (2000) and Iranian transplant organ procurement organization (2002). Obtaining the consent from the deceased donor families was based on a religious decree of the late Imam Khomeini (1989). The first living related liver transplantation was performed in 1996 and up to 2011, 165 cases have been transplanted.

The first split liver transplantation was done in 2002 and up to 2011, 56 cases have been transplanted. In his report, Dr. Malek-Hosseini mentioned that half of the transplanted livers and all of the transplanted kidneys from deceased donors were harvested from the South of Iran network. In Shiraz Transplant Center more than 80% of kidney transplant was performed from deceased donor since 2009.

The goal of this celebration was to glorify of deceased donors families. The ceremony was attended by a sizable number of families of organ donors and recipients. It was highlighted by emotional speech of recipients and donor families. Some of donors families, for the first time faced with the recipients who live with liver or kidney of their beloved ones, this confrontation was a unique occasion that impressed all participants.

In this ceremony, services of the special guest of the Congress, Professor J. Fung from Cleveland Clinic of USA, and the retired colleagues of Shiraz Organ Transplant Center were also appreciated.

